# Clinical Judgment of Home-Visit Nurses with an Extended Scope of Practice in Japan: A Qualitative Study

**DOI:** 10.3390/healthcare14203513

**Published:** 2026-10-19

**Authors:** Tomomi Gotoh, Masatoshi Saiki, Motoshi Ouchi, Ikuko Sakai

**Affiliations:** 1 Graduate School of Nursing, Chiba University, 1-8-1 Inohana, Chuo-ku, Chiba 260-8672, Japan; 2 Seikyo-Ukima Clinic, Tokyo-Hokuto Health Co-operative Association, 3-22-1 Ukima, Kita-ku, Tokyo 115-0051, Japan

**Keywords:** advanced practice nursing, clinical judgment, clinical reasoning, home-visit nursing, extended scope of practice, specified medical acts, Japan

## Abstract

**Highlights:**

This study qualitatively investigates the clinical judgment of home-visit registered nurses with an extended scope of practice (RNs-ESP) in Japan using participant ob-servation and semi-structured interviews. The resulting framework illustrates how these advanced practitioners integrate pathophysiological reasoning with patients’ daily lifestyles to optimize clinical interventions.

**What are the main findings?**

- The clinical judgment of RNs-ESP is structured into five core categories that highly integrate pathophysiological reasoning with patients’ daily lives: “Interpretation of pathological conditions and prediction of changes”, “Directing daily living assistance based on general conditions”, “Construction of the home care system in anticipation of pathological changes”, “Optimization of pharmacotherapy tailored to pathological conditions and daily life”, “Selection of medical procedures tailored to daily life”.
- Rather than merely implementing physicians’ instructions, RNs-ESP weigh quality of life against risks to balance clinical trade-offs and make decisions.

**What are the implications of the main findings?**

- The findings of this study could support healthcare administrators and physicians in constructing and improving organizational support systems and interprofessional collaborative practice models that facilitate the effective deployment and autonomous practice of RNs-ESP.
- The identified framework provides home-visit RNs-ESP with a practical tool for articulating and reflecting on clinical reasoning and for supporting professional education, peer learning, and the expanded development of their nursing roles.

**Abstract:**

**Background/Objectives:** The super-aged society and shortening of hospital stays in Japan have increased the importance of the role of home-visit nurses. The 2015 Specific Medical Acts Training Program qualified registered nurses with an extended scope of practice (RNs-ESP) to perform specified medical procedures based on physician-prepared protocols. Although the procedural activities of RNs-ESP have been documented, their underlying clinical judgments remain unclear. This study aimed to clarify the clinical judgments of home-visit RNs-ESP in Japan. **Methods:** This qualitative, descriptive study used participant observation and semi-structured interviews. Five home-visit RNs-ESP with at least 2 years of post-training experience participated. Data were collected from 32 clinical cases during home visits and analyzed using Mayring’s qualitative content analysis, utilizing Tanner’s clinical judgment model as a conceptual framework for operational definition. **Results:** The clinical judgments of RNs-ESP were classified into five categories and 18 subcategories: “Interpretation of pathological conditions and prediction of changes”, “Directing daily living assistance based on general conditions”, “Construction of the home care system in anticipation of pathological changes”, “Optimization of pharmacotherapy tailored to pathological conditions and daily life”, and “Selection of medical procedures tailored to daily life”. RNs-ESP integrated medical reasoning with patients’ life perspectives to design and implement optimal, highly individualized care plans. **Conclusions:** The clinical judgments of RNs-ESP are characterized by the integration of pathological assessment and daily life needs, supported by autonomous intervention authority. These judgments are essential nursing practices that balance continuous safe medical treatment with patient dignity and quality of life in home care.

## 1. Introduction

The number of home care patients with complex and diverse care needs is rapidly increasing in Japan owing to the super-aged society, the differentiation of hospital bed functions, and a reduction in the length of hospital stays [1,2,3]. Many of these home care patients have multimorbidity, which is characterized by multiple co-occurring chronic conditions, and are very dependent on medical care [3,4]. When visiting such highly individualized environments alone, home-visit nurses are required to make sequential clinical decisions while predicting and avoiding acute pathological changes and potential risks [5]. To make appropriate clinical judgments, nurses must comprehensively interpret a patient’s needs and health problems to determine the most appropriate actions [6]. Exercising such autonomous clinical judgment based on proactive foresight—predicting patients’ physical and psychological changes and risks—plays an extremely important role in the practice of home-visit nursing [7].

In 2015, Japan established the Specific Medical Acts Training Program for Nurses to enable trained nurses to undertake specified medical acts within defined protocols, thereby facilitating timely and appropriate care in home care settings [8]. Nurses who complete this training—hereafter referred to as registered nurses with an extended scope of practice (RNs-ESP)—exercise clinical judgment to perform specified medical procedures using physician-prepared “procedure manuals”, which serve as comprehensive pre-authorized instructions, without waiting for real-time instructions from a physician [8,9]. These procedures include initiating intravenous infusions for dehydration, performing wound debridement for intractable wounds, and replacing tracheal cannulas [8]. Although the number of trained nurses nationwide now exceeds 14,000, fewer than 10% of these actively work in home care settings, such as home-visit nursing stations [10]. Therefore, developing and disseminating concrete practice models in home care remains a major challenge for the whole nursing training system.

Since the inception of this system, studies have been conducted on how RNs-ESP nursing practice has changed following training [11]. However, to the best of our knowledge, most previous research has focused on the technical aspects of practice (i.e., which specified medical acts were performed). Specifically, what remains unclear is the comprehensive nature of the clinical judgments made by RNs-ESP prior to implementing protocols; namely, how they integrate the patient’s pathological condition with daily life information and what cognitive processes underlie their clinical decisions. Moreover, because home-visit nursing is conducted in isolated environments without the physical presence of a physician, the individual clinical judgments made by RNs-ESP remain invisible and tend to rely heavily on personal experiential knowledge, posing a substantial challenge to professional standardization.

Therefore, this study aimed to clarify the contents of clinical judgments made by home-visit RNs-ESP in Japan, focusing on the underlying thoughts and actions that guide their decision-making in home care settings.

In this study, clinical judgment was operationally defined as follows based on Tanner’s model [6] (p. 204): “An interpretation or conclusion about a patient’s needs, concerns, or health problems, and/or the decision to take action (or not), use or modify standard approaches, or improvise new ones as deemed appropriate by the patient’s response.”

## 2. Materials and Methods

### 2.1. Research Design

This study used a qualitative, descriptive research design, drawing on Yin’s exploratory and explanatory case study approach [12], to describe in detail the context-dependent phenomenon of clinical judgment made by RNs-ESP in Japanese home care settings.

### 2.2. Participants and Case Selection

Eligible participants met three primary inclusion criteria. First, nurses had to have completed the nationally approved Specific Medical Acts Training Program in Japan. Second, eligible candidates were required to be currently practicing as home-visit nurses in home-visit nursing stations or medical institutions and to have a minimum of 2 years of practical experience following program completion. This specific timeframe was selected based on previous literature indicating that advanced practice nurses typically require 6 months to 2 years to fully establish their clinical roles and practice models within an organization [13]. Third, the study placed no restrictions on the specific categories of completed medical procedures or additional professional qualifications, such as Certified Nurse or Nurse Practitioner.

Network sampling was used to recruit participants through professional home-visit nursing associations and designated training institutions in Japan. This strategy was necessary because RNs-ESP practicing in home care settings with at least 2 years of post-training experience constitute a small and highly specialized population. Potential candidates were contacted directly by the principal researcher, who explained the study purpose and obtained written informed consent prior to any research activities. The participating RNs-ESP were requested to designate observation dates that reflected their typical, routine daily home visits to ensure that the data represented actual clinical practice. The units of analysis were individual clinical cases, defined as all patients visited and cared for by the participating nurses over an observation period of 2 days per nurse. No encountered cases were excluded during this observation period.

The required sample size was determined by the criterion of data saturation during the qualitative content analysis. Data collection and analytical abstraction proceeded iteratively until data saturation was achieved, defined as the point at which continuous coding and comparative analysis of additional case dossiers yielded no new primary codes or subcategories.

### 2.3. Data Collection

Data were collected using a combination of participant observation and semi-structured interviews across the observed clinical cases. The participant observations captured real-time clinical encounters, while the subsequent semi-structured interviews and debriefings elucidated the underlying clinical reasoning and the rationale for RNs-ESP actions. The data collection period was from March to May 2024.

First, participant observation was conducted. The principal researcher accompanied each RN-ESP for 2 days during their working week, observing their clinical encounters in the patients’ homes. The researcher recorded the participants’ assessment behaviors, conversations with patients and families, and information sharing with physicians and multidisciplinary teams. To capture the underlying clinical reasoning and the rationale for participants’ actions, the researcher conducted brief, non-intrusive, face-to-face debriefings with the RNs-ESP during travel times or immediately before and after visits, lasting 10 to 20 min per case for a total of approximately 500 min. All observations and verbalized thoughts were recorded chronologically in field notes. To minimize observer bias, the researcher maintained field notes that strictly separated objective clinical actions from personal reflective impressions.

Second, following the completion of the participant observation phase, the researcher conducted a face-to-face, semi-structured interview with each RN-ESP using an interview guide. The interviews lasted approximately 60 min per participant for a total of approximately 300 min. Recall bias was minimized by conducting interviews on the same day as the observations, typically within 1 to 3 h after the home visits, and using specific observed clinical events as immediate prompts. The participants were asked to recall specific decisions and assessment behaviors observed during the home visits. They were prompted to explain why they chose certain actions, what physical or psychological changes they anticipated, and how they balanced the patient’s pathological conditions with their lifestyle and social factors. All audio recordings, totaling approximately 800 min across debriefings and interviews, were transcribed verbatim.

### 2.4. Data Analysis

The unit of analysis was each clinical case. The analytical data for each case integrated multiple data sources, including objective field notes from participant observations and debriefings, clinical documentation such as medical records and care plans, and verbatim interview transcripts. For each case, these data sources were compiled chronologically into a consolidated dossier. This integration enabled data triangulation, in which observational data captured actual clinical actions and environmental context, medical records provided objective clinical background, and interview transcripts elucidated the cognitive reasoning underlying the observed behaviors.

The data were analyzed using a predominantly inductive, qualitative content analysis based on Mayring’s framework [14] and informed by Tanner’s clinical judgment model [6] as a conceptual guide. Tanner’s framework was used to operationally define clinical judgment and guide the extraction of relevant decision-making segments from field notes and transcripts. The subsequent category formulation and code assignment proceeded inductively from the raw data following a stepwise process of abstraction.

Initial coding and initial category formulation were conducted by the principal researcher, T.G. To verify analytical consistency, all co-researchers (M.S., M.O., and I.S.) participated in the analytical review by repeatedly cross-checking the extracted contextual segments, primary codes, and evolving categories. Any interpretive disagreements about code assignment or category boundaries were thoroughly discussed during research team meetings until a full consensus was achieved. This process ultimately resulted in the alignment of all code interpretations.

The categories and subcategories were identified as follows. First, contextual segments describing what the RNs-ESP interpreted, anticipated, and decided were extracted based on the operational definition of clinical judgment. Second, formulated codes were created to preserve the linguistic nuances and contextual meanings of the participants’ narratives. Finally, iterative comparisons of the codes based on similarities and differences allowed for systematic categorization into subcategories and main categories.

### 2.5. Rigor and Trustworthiness

Analytical rigor and observational credibility were ensured by explicitly addressing the principal researcher’s background and reflexivity. The principal researcher is a primary care Nurse Practitioner in Japan whose doctoral research focused on clinical judgment. Although this clinical background facilitated rapid rapport and deep contextual understanding, it posed the challenge of potential bias. Reflexivity was maintained throughout the research process using data triangulation and peer debriefings.

The principal researcher ensured data accuracy by cross-referencing objective field notes from participant observations with interview transcripts and directly verifying the factual correctness of the recorded events and field note contents with the participating nurses during visits and immediate post-visit debriefings. Formal member checking was subsequently conducted by presenting draft case narratives, extracted primary codes, and the categorized clinical judgment structure directly to all five participating RNs-ESP. Confirmatory feedback was obtained from all five participants, who confirmed that the analytical categories accurately represented their actual clinical reasoning and practice, and did not require any structural modifications. Finally, the entire analytical process and category formulation were verified through peer debriefings with the co-researchers, who have extensive expertise in qualitative nursing methodology.

### 2.6. Ethical Considerations

This study was approved by the Institutional Review Board of the authors’ affiliated university. Prior to data collection, written and verbal explanations of the study purpose, voluntary nature of participation, freedom to withdraw at any time without penalty, and strict confidentiality of data were provided to all participating nurses, patients, and their family members. Signed consent was obtained from all participants. All data were pseudonymized and stored securely on a password-protected computer.

## 3. Results

### 3.1. Overview of Participants and Cases

A total of five RNs-ESP participated in this study, providing data across 10 days of participant observation and approximately 800 min of audio-recorded interviews. During these observation periods, 32 distinct clinical cases were analyzed.

Overall, data collection yielded approximately 800 min of audio recordings, combining 500 min from 32 post-visit debriefings and 300 min from 5 individual interviews. The detailed demographic profiles and clinical backgrounds of the five participating RNs-ESP are summarized in Table 1. Regarding the Specific Medical Acts Training Program, the completed training categories varied among participants according to their clinical specialties. Participants A, B, and C had completed targeted categories, such as wound management, surgical or peritoneal drain management, nutritional catheter management, and medication administration for nutrition and fluid management, whereas Participants D and E had completed all 21 categories, indicating completion of the full program.

The participating nurses were all female, aged in their 40s and 50s. Their overall nursing experience ranged from 12 to 34 years, with a mean of 19 years. Their home-visit nursing experience ranged from 4 to 14 years, with a mean of 9 years, and their practical experience as RNs-ESP ranged from 5 to 8 years, with a mean of 6 years. Their additional professional qualifications included one Certified Nurse in wound, ostomy, and continence care, one Certified Nurse in home care, and two Nurse Practitioners. Across the observation period, the 32 clinical cases were distributed among the participants as follows: Participants A and B contributed five cases each, Participant C contributed six cases, and Participants D and E contributed eight cases each (Table 1).

The patients in these analyzed cases were aged from their 30s to 90s. Regarding primary diagnoses, ten patients had terminal cancer and six had neurodegenerative diseases; the other cases presented with diabetic foot ulcers, heart failure, and dementia (Table S1). The analytical database established from these cases provided substantial qualitative depth, with data saturation confirmed as no additional primary codes or subcategories emerged during the analysis of the final cases.

The clinical judgments of RNs-ESP in this study involved the immediate integration of advanced pathophysiological and pharmacological assessments, protocol-based decision-making, and daily living care adjustments. Using expanded knowledge gained through specified medical acts training, the participating nurses differentiated complex clinical symptoms at the bedside, determined the immediate scope of medical interventions using procedure manuals, and aligned daily care to ensure patient safety and quality of life (QOL).

The clinical judgments made by the RNs-ESP were classified into five main categories and 18 subcategories. Table 2 presents the overarching structural framework of these main categories and subcategories derived from the qualitative content analysis. All 18 subcategories were derived from and supported by observational and interview data across multiple participants and cases; representative narrative case vignettes illustrating each subcategory are presented in Section 3.2. Qualitatively, all five main categories of clinical judgment were consistently represented across all five participants, demonstrating that the overarching framework captures core clinical judgment dimensions common to home-visit RNs-ESP regardless of individual practice settings.

### 3.2. Interpretation of Pathological Conditions and Prediction of Changes

In the following narrative descriptions, representative case excerpts and quotes are presented to illustrate the contextual nuances of each subcategory. Although individual case vignettes are cited as typical examples, each subcategory was fundamentally grounded in recurring patterns of clinical reasoning observed across multiple cases and participants.

RNs-ESP actively interpreted pathological mechanisms from physical signs and vital signs, proactively anticipating disease progression, potential complications, and signs of end-of-life deterioration.

#### 3.2.1. Reasoning of the Causes and Potential Effects of Symptoms

RNs-ESP differentiated the underlying factors of physical distress and evaluated how these symptoms affected the psycho-social lives of patients and their families.

*“Regarding a terminal cancer patient showing extreme restlessness, I wonder if he is suffering now or if he would be more comfortable sleeping. But increasing the medication further risks respiratory depression, so sedating him isn’t the right answer either. If the cause of his nighttime restlessness is residual urine, his inability to void makes him fidgety. This is painful not only for him but also for his family watching him, who can’t sleep. I should check for residual urine and perform catheterization if necessary.”*

(Case 17)

#### 3.2.2. Prediction of Disease Progression Based on Chronological Physical Findings

By comparing physical findings over time, RNs-ESP evaluated the efficacy of current treatments and assessed the feasibility of weaning patients off medical devices.

*“The range and sound of crackles are clearly better than yesterday. His breathing after using the toilet has also settled down, and the edema has decreased. The diuretic has finally started to work, so I decided he could manage without oxygen.”*

(Case 8)

#### 3.2.3. Prediction of Complications in Anticipation of Reduced Physiological Reserve

RNs-ESP assessed the decline in physiological reserve owing to aging or disease progression to anticipate and mitigate secondary risks like pneumonia, dehydration, or constipation.

*“In this condition, there are no visible signs of pneumonia, but it could easily occur. To maintain his physiological reserve, we must monitor his nutrition and weight. It’s difficult for him to regulate his body temperature, so heat builds up, which means he is prone to dehydration and his constipation will likely worsen. I keep these future things in mind when planning.”*

(Case 29)

#### 3.2.4. Identification of End-of-Life Timing Through the Interpretation of Vital Signs

RNs-ESP evaluated chronological changes in vital signs and physical appearance to assess impending end-of-life deterioration, enabling timely communication with family members and physicians about palliative care comfort measures.

*“On the evening of the day before yesterday, his breathing pattern changed to mandibular breathing, SpO_2_ was in the 80s, blood pressure was in the 60s, and his pupils were slightly dilated. I knew he had only a few hours left. I conveyed to the doctor that he was in the very terminal stage, and it wouldn’t be surprising if he passed away within hours.”*

(Case 19)

### 3.3. Directing Daily Living Assistance Based on General Conditions

RNs-ESP assessed physical functions to determine the suitability and direction of daily living assistance, balancing functional maintenance with patient safety.

#### 3.3.1. Selection of Living Environments to Compensate for Physical Limitations

RNs-ESP evaluated whether motor difficulties were side effects of medications or resulted from muscle/sensory decline, and adjusted the patient’s environment accordingly.

*“Regarding her upper limb tremors, her horizontal movement is fine. It doesn’t seem to be a drug side effect; rather, it is a muscle strength issue. She also has poor vision and struggles with depth perception. I adjusted the desk height and dish positions so she can eat more easily.”*

(Case 6)

#### 3.3.2. Determination of Safe Activity Ranges Based on an Analysis of Pain-Related Factors

RNs-ESP reasoned about the multifactorial causes of pain to balance pharmacological and non-pharmacological interventions.

*“I feel this back pain is not just somatic. She has spinal canal stenosis and numbness, and the pain at the start of movement is probably influenced by muscle tension from inactivity. If so, we need more than just opioids; some movement and stretching are necessary.”*

(Case 32)

#### 3.3.3. Determination of Alternative Hygiene Care Considering Infection Risks

RNs-ESP balanced patients’ hygiene requests with infection risks during critical treatment phases, choosing safer alternative care methods.

*“I know he wants to take a bath, but because of maintenance debridement, his wound bed is exposed. It has entered the granulation phase, so I want it to heal as soon as possible without infection complications. Therefore, I postponed the bath and decided to perform a bed bath instead.”*

(Case 5)

### 3.4. Construction of the Home Care System in Anticipation of Pathological Changes

RNs-ESP anticipated the illness trajectory to support patient/family decision-making and pre-emptively structured collaborative care systems.

#### 3.4.1. Planning of Decision-Making Support Considering Cognitive Functions and Emotions

RNs-ESP explored the feelings underlying the behaviors of patients with cognitive decline to establish stepwise, stress-free decision-making support.

*“Because of her dementia and higher brain dysfunction, it’s hard to understand what she is thinking. However, her words and thoughts are just disconnected. If I speak to her empathetically, she can express agreement or disagreement. When discussing important things about the future, I must ensure she fully understands and consents to one suggestion before moving to the next.”*

(Case 6)

#### 3.4.2. Selection of Care Locations Balancing Prognosis and Family Intentions

For dying patients, RNs-ESP assessed whether the family could cope with home care, and made decisions about the feasibility of fully transitioning to end-of-life care at home.

*“His prognosis was down to days. Although he could have been admitted to the reserved palliative care ward, moving him now when the family is comfortable with home caregiving felt wrong. I told the family that we should support them so they can be with him and have peace of mind when he passes away at home.”*

(Case 17)

#### 3.4.3. Reallocation of Medical Resources According to Aggravation Risks

For patients with complex multimorbidity, RNs-ESP evaluated the need to reallocate home medical resources to maintain safety.

*“His diabetes is stable, but he has anemia and struggles to breathe, so he needs a thorough examination. The doctor suggested an online consultation since the outpatient specialist is seeing him. But we must establish a proper system for general physical management, so I requested an in-person home visit.”*

(Case 10)

### 3.5. Optimization of Pharmacotherapy Tailored to Pathological Conditions and Daily Life

When using protocols for medication adjustment, RNs-ESP evaluated the medication’s impact on daily life and designed optimal administration plans.

#### 3.5.1. Evaluation of Prescription Validity Balancing QOL and Side Effects

RNs-ESP weighed the benefits of drug efficacy against side effects that compromise daily living, continuously assessing prescription validity.

*“When starting mirogabalin for foot pain, I worried that while the pain might decrease, drowsiness could prevent him from eating. Being sleepy means the pain is relieved, which is good. But if he can’t eat, it’s a problem. I will monitor if the dosage is appropriate so the home helper won’t feel anxious.”*

(Case 8)

#### 3.5.2. Determination of Prescription Contents to Break the Vicious Cycle of Symptoms

RNs-ESP identified the potential risk of secondary complications, such as peptic ulcers or dehydration, caused by persistent symptoms like vomiting and proposed preemptive prescription changes to doctors.

*“Our current challenge is vomiting. Continuous vomiting can inflame the esophagus and lead to peptic ulcers, worsening her nausea and appetite. I proposed trying an antiemetic to break this vicious cycle.”*

(Case 12)

#### 3.5.3. Redesign of Administration Plans Considering Caregiving Burden

RNs-ESP balanced standard clinical recommendations, such as thrice-daily intravenous infusions, with the family’s caregiving capacity, redesigning plans to be manageable within the domestic context.

*“He has a fever, and this is likely cellulitis. Ideally, he needs thrice-daily IV drip management, but the family’s capacity is once a day. I will talk to the doctor about starting with injections and then transitioning to oral meds, adjusting the dosage for his renal function.”*

(Case 10)

#### 3.5.4. Determination of the Need for Fluid Therapy to Improve End-of-Life Comfort

For terminally ill patients unable to swallow, RNs-ESP decided whether to initiate subcutaneous hydration based on the protocol, aiming to relieve symptoms and reassure the family rather than extend the patient’s life.

*“He has managed to eat until now, but in the past week he has barely eaten. He previously refused a gastrostomy tube. However, providing a small amount of fluid will make his body more comfortable and help give the family peace of mind. The protocol is ready, so it’s time to initiate hydration.”*

(Case 23)

### 3.6. Selection of Medical Procedures Tailored to Daily Life

In managing wounds and medical devices, RNs-ESP integrated local clinical findings with the patient’s QOL and the caregivers’ burden to select procedures.

#### 3.6.1. Selection of Non-Invasive Care Considering Treatment Policies and Life Constraints

RNs-ESP aligned procedures with the overall goal of healing versus maintenance, selecting minimally invasive approaches to reduce patient distress and caregiver burden.

*“For foot gangrene from arteriosclerosis, the goal is not active healing but dry maintenance while preventing infection. Since we visit daily and active checking would only increase the care staff’s anxiety and constrain the patient, I decided to stop complex wound markings that demand extra manpower.”*

(Case 8)

#### 3.6.2. Selection of Procedures to Assess General Conditions and Local Healing Stages

RNs-ESP connected local wound issues with systemic pathology, such as the effects of anticoagulant therapy, diabetes, or edema to determine the suitability and timing of invasive procedures.

*“He is on anticoagulants for deep vein thrombosis, so he bleeds easily and his wounds heal slowly. However, maintenance debridement was necessary, so we continued it. Finally, the wound bed is exposed, and vascularization is rich, meaning it has entered the granulation phase. We can safely suspend debridement now.”*

(Case 5)

#### 3.6.3. Decision to Modify Medical Devices According to Pathological Transitions and QOL

RNs-ESP evaluated how device modifications would affect functional recovery and motivation, choosing devices that improve QOL.

*“Recently, he has been swallowing his saliva well. With the current cuffed tracheostomy tube, he can’t speak and feels uncomfortable. I decided to change to a cuffless speaking cannula. This will boost his motivation for rehabilitation.”*

(Case 28)

#### 3.6.4. Directing Preventive Interventions Anticipating the Risks of Long-Term Medical Device Placement

To mitigate risks like granulation or bleeding from long-term tubes, RNs-ESP planned and implemented stepped, preventive interventions.

*“The granulation around the gastrostomy tube is relatively clean, but there was minor bleeding during replacement. We are using a strong topical steroid, but for the next replacement, I will shorten the shaft length to secure it tightly. If that doesn’t work, we’ll arrange for chemical cauterization.”*

(Case 29)

## 4. Discussion

The results of this study indicate that the clinical judgments of participating home-visit RNs-ESP are based on interpreting pathological conditions and predicting changes. These judgments range from holistic daily-life coordination and care-system design (such as directing daily living assistance based on general conditions and constructing the home care system in anticipation of pathological changes) to highly specific clinical and physical interventions, including the optimization of pharmacotherapy tailored to pathological conditions and daily life and the selection of medical procedures tailored to daily life. While the former categories reflect foundational home-visit nursing competencies augmented by physical assessment, the latter categories highlight judgment dimensions specific to the extended-scope practice context, in which RNs-ESP exercise protocol-based clinical discretion.

### 4.1. Integration of Medical Reasoning and Daily Life Perspective

The clinical judgments of participating RNs-ESP observed in this study were characterized by the seamless synthesis of advanced medical pathology and daily living contexts. Because this study did not include a comparator group of conventional home-visit nurses, comparison of the findings with those from traditional nursing practice is framed conceptually by reference to previous literature rather than by direct empirical comparison. In the published literature, conventional home-visit nursing practice is generally described as focusing on holistic living support, patient-centered care, and the early detection of physical changes to report to physicians [15]. Interpreted through this literature-based lens, our findings suggest that participating RNs-ESP build upon and extend these foundational nursing competencies by applying pathophysiological and pharmacological reasoning to trace physical symptoms back to underlying mechanisms. This reasoning was empirically observed among participants in judgment categories such as the optimization of pharmacotherapy tailored to pathological conditions and daily life, as well as the selection of medical procedures tailored to daily life. Supported by procedure manuals, RNs-ESP in our sample did not merely observe and report abnormalities; instead, they exercised protocol-supported discretion to execute or propose immediate medical adjustments, such as fluid management and medication optimization, while tailoring daily living assistance. This foresight appeared to enable participating nurses to anticipate future deterioration and proactively modify care environments within their professional scope [7].

### 4.2. Optimization of Care and Autonomy Under Protocol Discretion

In Western countries, Advanced Practice Nurses or Nurse Practitioners typically have autonomous prescriptive authority and independent practice rights [16]. In contrast, Japanese RNs-ESP operate within a highly controlled regulatory framework, implementing specified medical procedures strictly in accordance with physician-prepared procedure manuals (protocols) [8,9]. Despite these structural constraints, the categories of “Optimization of pharmacotherapy tailored to pathological conditions and daily life” and “Selection of medical procedures tailored to daily life” suggest that participating RNs-ESP do not simply perform mechanical tasks or seek “medical perfection” at the cost of the patient’s lifestyle. Instead, they balance standard clinical recommendations against the patient’s QOL, family caregiving capacity, and environmental constraints. Supported by their advanced medical training, participating RNs-ESP evaluate how far a treatment can be modified, delayed, or suspended safely within protocol boundaries. This clinical reasoning process supports the design of individualized care plans that respect patient values and domestic life.

### 4.3. Restructuring Team Medicine Through Collaborative Clinical Judgment

Rather than making decisions in isolation or acting as simple task delegates, RNs-ESP in this study frequently engaged in collaborative decision-making with physicians. This collaborative approach aligns with interprofessional collaboration frameworks in which clinical roles and responsibilities are negotiated dynamically to optimize patient care [17]. Instead of relying on a traditional hierarchical or unilateral physician–nurse relationship [18], participating RNs-ESP integrated comprehensive physical assessments within the patient’s domestic context to propose targeted medication adjustments or procedure modifications, which physicians subsequently approved or refined. This collaborative clinical judgment suggests that participating RNs-ESP do not function merely to shift tasks from physicians, aligning with recently proposed organizational frameworks for the utilization and deployment of RNs-ESP [19]. When interpreted through external theoretical frameworks, such as that proposed by Mayo et al. [20], fostering psychological safety is a foundational prerequisite for facilitating open communication and maximizing collective intelligence within interprofessional teams. However, because our qualitative findings mainly reflect collaborative decision-making between RNs-ESP and physicians rather than the broader interdisciplinary team, this theoretical link remains a plausible hypothesis. Future empirical research is needed to evaluate how this collaborative model influences interactions with other team members, such as care managers and rehabilitation specialists, and whether it objectively elevates team decision-making quality in home care.

### 4.4. Limitations

Several methodological limitations of this study should be noted. First, the small sample size of five experienced RNs-ESP recruited via network sampling in specific regions and the lack of a control group may have introduced selection bias, limiting direct generalizability to novice nurses, other healthcare settings, or broader quantitative verification. Second, the observational data were collected over a brief period of 2 days per nurse across multiple cases, which may not have fully eliminated the Hawthorne effect or the influence of the researcher’s presence on participant behavior during field visits. Third, contextual influences—such as attending physician relationships, organizational climate, and disease-specific trajectories across the heterogeneous 32 cases—were not directly measured or controlled, leaving their specific impact beyond the empirical scope of this study.

### 4.5. Future Directions

To address these limitations, future research should employ quantitative and multi-center designs with larger cohorts to externally validate the identified clinical judgment framework. Additionally, studies focusing on disease-specific cohorts are recommended to examine how these core judgment processes are tailored to particular clinical trajectories. Further research is also warranted to investigate how organizational support frameworks and collaborative practice models affect the implementation of RN-ESP clinical judgment and to evaluate objective patient outcomes, such as the rates of avoidable hospitalization and QOL.

## 5. Conclusions

This qualitative study provides preliminary contextual insights into the clinical judgment of home-visit RNs-ESP in Japan. The resulting framework offers a practical reference for home-visit nurses to articulate and reflect on their clinical reasoning. Further quantitative research with larger cohorts is needed to evaluate the broader applicability and clinical outcomes of this practice model.

## Figures and Tables

**Table 1:** Characteristics of participating RNs-ESP and observed cases.

ID	Nursing Experience (Years)	Home-Visit Nursing Experience (Years)	RNs-ESP Experience (Years)	Additional Professional Qualifications	Completed Specific Medical Act Training Categories	Number of Cases
A	34	8	5	Certified Nurse in Wound, Ostomy and Continence Nursing	Wound Management, Surgical Wound Drain Management, Medication Administration for Nutrition and Fluid Management	5
B	14	10	6	None	Wound Management, Surgical Wound Drain Management, Peritoneal Drain Management, Nutritional Catheter Management	5
C	17	14	8	Certified Nurse in Home Care	Medication Administration for Nutrition and Fluid Management	6
D	19	4	8	Primary Care Nurse Practitioner	All 21 categories	8
E	12	7	5	Primary Care Nurse Practitioner	All 21 categories	8

RNs-ESP, registered nurses with an extended scope of practice.

**Table 2:** Categories and subcategories of the contents of clinical judgment.

Main Categories	Subcategories
Interpretation of pathological conditions and prediction of changes	▪ Reasoning of the causes and potential effects of symptoms ▪ Prediction of disease progression based on chronological physical findings ▪ Prediction of complications in anticipation of reduced physiological reserve ▪ Identification of end-of-life timing through the interpretation of vital signs
Directing daily living assistance based on general conditions	▪ Selection of living environments to compensate for physical limitations ▪ Determination of safe activity ranges based on an analysis of pain-related factors ▪ Determination of alternative hygiene care considering infection risks
Construction of the home care system in anticipation of pathological changes	▪ Planning of decision-making support considering cognitive functions and emotions ▪ Selection of care locations balancing prognosis and family intentions ▪ Reallocation of medical resources according to aggravation risks
Optimization of pharmacotherapy tailored to pathological conditions and daily life	▪ Evaluation of prescription validity balancing QOL and side effects ▪ Determination of prescription contents to break the vicious cycle of symptoms ▪ Redesign of administration plans considering caregiving burden ▪ Determination of the need for fluid therapy to improve end-of-life comfort
Selection of medical procedures tailored to daily life	▪ Selection of non-invasive care considering treatment policies and life constraints ▪ Selection of procedures to assess general conditions and local healing stages ▪ Decision to modify medical devices according to pathological transitions and QOL ▪ Directing preventive interventions anticipating the risks of long-term medical device placement

QOL, quality of life.

## Data Availability

The data presented in this study are available on request from the corresponding author due to privacy and ethical restrictions regarding qualitative data (raw transcripts and field notes).

## Supplementary Materials

The following supporting information can be downloaded at: https://www.mdpi.com/article/10.3390/healthcare14203513/s1, Table S1. Demographic and clinical profiles of the analyzed patient cases.

## Abbreviations

The following abbreviations are used in this manuscript:

QOL	Quality of Life
RNs-ESP	Registered Nurses with an Extended Scope of Practice
