# Supplementary material for: Clinical Judgment of Home-Visit Nurses with an Extended Scope of Practice in Japan: A Qualitative Study
Source: Healthcare (Basel). 2026 Oct 19;14(20):3513. doi: 10.3390/healthcare14203513 (PMC13704268; doi:10.3390/healthcare14203513)
Supplement: Supplementary file 1 [file healthcare-14-03513-s001.zip › healthcare-4574300-supplementary.pdf]

healthcare

Table S1. Demographic and Clinical Profiles of the Analyzed Patient Cases.

No.	Age Group	Gender	Primary Diagnosis / Presenting Clinical Condition
1	80s	Male	Interstitial pneumonia
2	80s	Male	Postoperative pneumatosis intestinalis
3	80s	Female	Terminal bladder cancer
4	50s	Male	Post-cerebral infarction
5	70s	Female	Terminal endometrial cancer
6	70s	Female	Terminal endometrial cancer
7	80s	Male	Terminal gastric cancer
8	90s	Female	Arteriosclerosis obliterans
9	70s	Male	Myelodysplastic syndrome
10	80s	Female	Cellulitis
11	70s	Male	Chronic heart failure and chronic obstructive pulmonary disease
12	30s	Female	Cerebral palsy
13	60s	Male	Intractable lower leg wound
14	50s	Male	Multiple sclerosis
15	80s	Female	Parkinson’s disease
16	80s	Male	Terminal lung cancer
17	60s	Male	Terminal sigmoid colon cancer
18	70s	Male	Amyotrophic lateral sclerosis
19	90s	Female	Terminal hepatocellular carcinoma
20	90s	Female	Terminal gastric cancer
21	90s	Female	Parkinson’s disease
22	80s	Female	Neurogenic bladder
23	90s	Female	Aspiration pneumonia
24	50s	Male	Amyotrophic lateral sclerosis
25	80s	Female	Parkinson’s disease
26	70s	Male	Multiple system atrophy
27	50s	Female	Anxiety disorder
28	70s	Male	Post-cerebellar hemorrhage
29	50s	Female	Spinocerebellar degeneration
30	70s	Male	Post-cerebral infarction
31	40s	Male	Schizophrenia
32	60s	Male	Diabetic foot ulcer
